# Development and Evaluation of a Water-Free In Situ Depot Gel Formulation for Long-Acting and Stable Delivery of Peptide Drug ACTY116

**DOI:** 10.3390/pharmaceutics16050620

**Published:** 2024-05-05

**Authors:** Yingxin Xiong, Zhirui Liu, Yuanqiang Wang, Jiawei Wang, Xing Zhou, Xiaohui Li

**Affiliations:** 1 Institute of Materia Medica and Department of Pharmaceutics, College of Pharmacy, Army Medical University, Chongqing 400038, China; yx.xiong@gmail.com; 2 Department of Pharmacy, Xinan Hospital, Army Medical University, Chongqing 400038, China; zhirui_liu@tmmu.edu.cn; 3 Chongqing School of Pharmacy and Bioengineering, Chongqing University of Technology, Chongqing 400054, China; wangyqnn@cqut.edu.cn (Y.W.); wangjiawei0423@gmail.com (J.W.); 4 Yunnan Key Laboratory of Stem Cell and Regenerative Medicine, Science and Technology Achievement Incubation Center, Kunming Medical University, Kunming 650500, China; 5 Engineering Research Center for Pharmacodynamics Evaluation, College of Pharmacy, Army Medical University, Chongqing 400038, China

**Keywords:** peptide, in situ depot gel, long-acting injectable, stability, molecular dynamics

## Abstract

In situ depot gel is a type of polymeric long-acting injectable (pLAI) drug delivery system; compared to microsphere technology, its preparation process is simpler and more conducive to industrialization. To ensure the chemical stability of peptide ACTY116, we avoided the use of harsh conditions such as high temperatures, high shear mixing, or homogenization; maintaining a water-free and oxygen-free environment was also critical to prevent hydrolysis and oxidation. Molecular dynamics (MDs) simulations were employed to assess the stability mechanism between ACTY116 and the pLAI system. The initial structure of ACTY116 with an alpha helix conformation was constructed using SYBYL-X, and the copolymer PLGA was generated by AMBER 16; results showed that PLGA-based in situ depot gel improved conformational stability of ACTY116 through hydrogen bonds formed between peptide ACTY116 and the components of the pLAI formulation, while PLGA (Poly(DL-lactide-co-glycolide)) also created steric hindrance and shielding effects to prevent conformational changes. As a result, the chemical and conformational stability and in vivo long-acting characteristics of ACTY116 ensure its enhanced efficacy. In summary, we successfully achieved our objective of developing a highly stable peptide-loaded long-acting injectable (LAI) in situ depot gel formulation that is stable for at least 3 months under harsh conditions (40 °C, above body temperature), elucidating the underlying stabilisation mechanism, and the high stability of the ACTY116 pLAI formulation creates favourable conditions for its in vivo pharmacological activity lasting for weeks or even months.

## 1. Introduction

Peptides are a unique class of molecules that show diverse biological activities, rendering them appealing for therapeutic applications. Insulin, the pioneering peptide therapeutic developed for diabetes treatment [1], approved for medical use in the early 1920s, has been playing a pivotal role in diabetes management [2]. The success of insulin paved the way for peptide therapeutics, yet challenges persist. Peptides are susceptible to protease degradation in the gastrointestinal tract, limiting their oral bioavailability, and their size and hydrophilicity hinder cellular membrane penetration, often necessitating injection. Moreover, rapid renal clearance and enzymatic degradation lead to short half-lives, requiring frequent administration and incurring compliance and cost issues. Peptides also face chemical and conformational instability, compromising their efficacy and safety [3,4]. Formulating stable and long-acting peptide dosage forms is a complex task.

**Figure 1:**
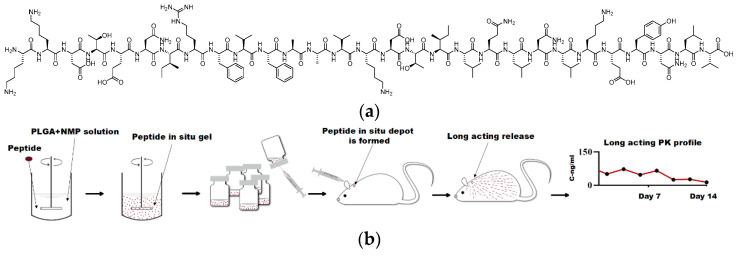
(**a**) Structure of peptide ACTY116; (**b**) schematic illustration of in situ depot gel preparation and in vivo long-acting performance.

Cardiac hypertrophy is a common cardiac disease characterised by an increase in cardiomyocyte volume and ventricular wall thickness, impairing cardiac function, which may include chest pain, dyspnea, syncope, arrhythmia, etc., and may even lead to heart failure or sudden death in severe cases. ACTY116 is a peptide designed to mimic the carboxyl terminus of Gαq protein [5,6], which can inhibit Gαq-mediated signal transduction and attenuate or reverse cardiac hypertrophy induced by pressure overload. ACTY116 has advantages such as high activity, low toxicity, and multiple effects, making it a promising candidate for anti-cardiac hypertrophy therapy [7,8]. As a 29-amino acid peptide (MW: 3424, its structure is depicted in Figure 1a), it faces the same challenges as other peptides, with extremely low oral bioavailability, poor stability, and a short in vivo half-life. Considering that cardiac hypertrophy is a chronic disease that requires long-term treatment, the objective of this study was to develop a stable long-acting injectable (LAI) formulation of peptide ACTY116 for sustained delivery.

The polymeric long-acting injectable (pLAI) formulations based on biodegradable polymers are widely utilised to extend the half-life of an active pharmaceutical ingredient (API). These formulations primarily encompass two forms: microspheres and in situ depot gels, with microspheres being the most extensively researched [9,10,11,12,13]. Approved microsphere LAIs for peptide delivery can exhibit prolonged in vivo residence time; Lupron Depot (leuprorelin acetate), Sandostatin LAR Depot (octreotide acetate), and Trelstar (triptorelin pamoate) are good examples [14,15]. Peptides are chemically unstable molecules, and achieving long-term stability at body temperature (37 °C) over several months poses a significant challenge. Thus, this study aimed to design, screen, and optimise a pLAI formulation capable of stabilising peptide ACTY116 for extended release in the human body.

Given the inherent susceptibility of peptide molecules to hydrolysis, this study aimed to develop a water-free in situ depot gel formulation [16,17] for ACTY116, and its stability was compared to that of ACTY116 microspheres. Stability assessments were conducted under challenging (40 °C, above body temperature) and long-term storage (25 °C) conditions, and in vitro release, in vivo pharmacokinetics, and pharmacological efficacy of ACTY116pLAI in situ depot gel were also evaluated (Figure 1b).

## 2. Materials and Methods

### 2.1. Materials

ACTY116 was synthesised by a contract research organization (HLXK, Beijing, China), PLGA was purchased from Evonik (Darmstadt, Germany) and Tanshtech (Guangzhou, China), N-methylpyrrolidone (NMP) and dichloromethane (DCM) were purchased from Chengdu Kelong Chemical Co., Ltd. (Chengdu, China), mannitol was purchased from Roquette (Lestrem, France), the NT-proBNP enzyme-linked immunosorbent assay (ELISA) kit was purchased from Elabscience Biotechnology (Wuhan, China), the BNP and β-MHC antibody immunohistochemical kit and diaminobenzidine (DAB) chromogenic agent were purchased from Servicebio (Wuhan, China), and norepinephrine (NE) was purchased from Sigma (Shanghai, China).

The moist heat autoclave (XD1-D, Xinhua, Shandong, China), IKA digital mixer (RW20, IKA, Staufen, Germany), polarizing microscope (BK-POL, Aote Optical Instrument, Chongqing, China), thermostatic oscillator (SHA-C, GY2016-SW, Changzhou, China), digital rotary viscometer (NDJ-8S, Fangrui, Shanghai, China), laser diffraction particle size analyzers (Mastersizer 3000, Malvern, Worcestershire, UK), ultra-high performance liquid chromatograph (e2695-2998, Waters, Milford, MA, USA), LC-MS/MS (6460, Agilent, Santa Clara, CA, USA), refrigerated centrifuge (Legend Micro 17R, Thermo, Waltham, MA, USA), microplate reader (infinite M200 pro, Tecan, Grödig, Austria), slide scanner (3D HISTECH, Budapest, Hungary), and Leica microscope (DM500, Leica, Wetzlar, Germany) were also procured.

### 2.2. Animals

BALB/C mice (20–25 g) were purchased from Army Medical University, China; SD rats (200–250 g) were purchased from Ensiweier Biotechnology, China. All animals had free access to a standard diet and drinking water and were housed in a room maintained at 22.0 ± 3 °C and with a 12:12 h cyclic lighting schedule. All animal experiments were approved by Laboratory Animal Welfare and Ethics Committee of Army Medical University (approval no.: AMUWEC20203377), and all the experiments were performed in accordance with the National Institutes of Health guidelines for the care and use of laboratory animals.

### 2.3. Method

#### 2.3.1. Preparation of Different Dosage Forms

##### Preparation of ACTY116 Solution

Two milligrams (2 mg) of ACTY116 were weighed and added into 20 mL saline solution, stirring till dissolved (pH was 5.35), and ACTY116 saline solution was filled into vials (F1).

##### Preparation of ACTY116 PLGA Microspheres

**Figure 2:**
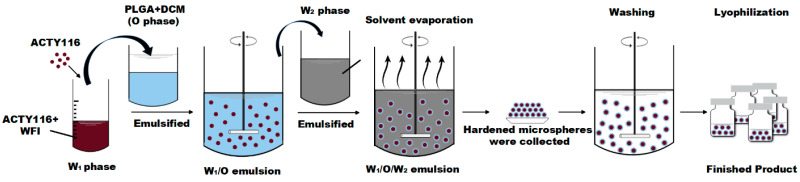
Schematic illustration of double emulsion–solvent extraction/evaporation technique for microsphere preparation.

The double emulsion–solvent extraction/evaporation method (Figure 2) was adopted due to the high hydrophilicity of ACTY116 [18]. ACTY116 (2 mg) was weighed and dissolved in 200 µL of water for injection (WFI) as water phase (W1 phase), 300 mg of PLGA (with a ratio of lactide to glycolide at 50:50, MW:7000–17,000, acid-terminated) was dissolved in 2 mL of DCM as oil phase, and a hydrophilic drug solution (W_1_ phase) was emulsified in the organic polymer solution (O phase) under 15,000 rpm high shear mixing for 5 min to form the primary water-in-oil (W_1_/O) emulsion. The obtained W_1_/O emulsion was subsequently added into 200 mL of 1% PVA solution (W_2_ phase) under 25,000 rpm high shear mixing for 10 min to form double emulsion (W_1_/O/W_2_). Evaporation of DCM was performed under 50 ± 1 °C, 100 rpm continuous shaking condition for 4 h, the microsphere suspension was centrifuged at 5000 rpm for 2 min (4 °C), and then dispersed with 10 mL of WFI for washing PVA and the unencapsulated ACTY116. The washing step was repeated for 5 cycles. Finally, the microspheres were dispersed in 10% mannitol solution, filled into vials, and lyophilised (F2) with the process as below (Table 1).

##### Evaluation of Microspheres

ACTY116 microspheres were dispersed in 10 mL of purified water to obtain a suspension, which was dropped onto the glass slide for polarising microscopic observation. Particle size was determined using laser diffraction particle size analysers, in which 2 mL of suspension was dispersed in 250 mL purified water, and instrument parameters were set to background measurement duration for 10 s, sample measurement duration for 30 s, obscuration range as 5.0–10.0%, and stirrer speed at 1000 rpm.

Encapsulation efficiency (EE): 40 mg of microspheres were weighed into a 5 mL centrifuge tube. Subsequently, 4 mL of purified water was added, and the mixture was shaken to ensure uniform dispersion of the microspheres. After centrifuging at 10,000 rpm for 5 min, the supernatant was collected to measure unencapsulated peptide. The microspheres that had settled at the bottom were transferred to a 25 mL volumetric flask using DMSO, the centrifuge tube was rinsed with DMSO for five times, and the rinses were pooled in the same volumetric flask; the mixture was then diluted to volume and thoroughly mixed, and the encapsulated peptide (ACTY116) was quantified using HPLC. Encapsulation efficiency was calculated using the following expression:

(1) $$\mathrm{Encapsulation}\;\mathrm{efficiency}\;(\mathrm{EE})\%=\frac{\mathrm{Encapsulated}\;\mathrm{peptide}}{\mathrm{Unencapsulated}\;\mathrm{peptide}+\mathrm{Encapsulated}\;\mathrm{peptide}}\times 100\%$$

##### Preparation of ACTY116 pLAI In Situ Depot Gel

Following an extensive review of the literature, we designed the pLAI in situ depot gel formulation using PLGA (with a ratio of lactide to glycolide at 50:50, MW:7000–17,000, acid-terminated) as the sustained-release matrix [9,17,19,20]. An amount of 660 mg of PLGA was first dissolved into 1280 mg of NMP, and then 56 mg of ACTY116 was added and stirred for about 10 min until a suspension was visually observed, and the suspension was filled into vials for use (F3).

#### 2.3.2. Stability Evaluation of Different Dosage FormFs

##### Assay Method for ACTY116

In the HPLC assay method for ACTY116, a C18 chromatographic column (150mm × 4.6 mm, 5 μm) was utilised, and the analysis was performed at a wavelength of 220 nm, maintaining a column temperature of 40 °C, and a constant flow rate of 1 mL/min was employed, with an injection volume of 5 μL. The gradient elution process is shown below (Table 2), with the mobile phase transitioning from A (water and 0.1% trifluoroacetic acid) to B (acetonitrile and 0.1% trifluoroacetic acid) over a specified time period.

##### Chemical Stability for Different Dosage Forms

To investigate the chemical stability of ACTY116 during the preparation of different dosage forms, assay values were analysed and compared between different stages of preparation, and a short-term stability was performed under 40 °C for 5 days and 10 days.

##### Determination of Secondary Structure of ACTY116 Using Circular Dichroism

The secondary structure of ACTY116 before and after preparation in different dosage forms (solution (F1), microspheres (F2), and pLAI in situ depot gel (F3)) were tested, and a short-term secondary structure stability under 40 °C for both 5 days and 10 days were also tested, respectively. Samples were diluted to 0.05 mg/mL of ACTY116 and then transferred into the quartz cell (1 mm) for a circular dichroism (CD) spectra measurement, which was carried out on a computer-assisted Chirascan qCD circular dichroism spectrometer. The CD spectra were recorded from 185 to 280 nm with bandwidth of 1 nm and step size of 1 nm.

#### 2.3.3. Formulation Optimization of ACTY116 pLAI In Situ Depot Gels

Acid-terminated PLGA (with a ratio of lactide to glycolide at 50:50, MW: 7000–17,000) was used in the study.

Evaluation of the impact of NMP on ACTY116 stability in pLAIs was performed by varying the ratio of NMP to ACTY116, and the formulations are provided in Table 3. The different formulations were filled into 7 mL vials and sealed with stoppers, respectively. The filled vials were autoclaved under 121 °C for 8 min and then tested for assay value by a HPLC method.

Evaluation of the impact of PLGA quantity on ACTY116 stability in pLAIs was also performed by varying the ratio of PLGA to ACTY116, and the formulations are provided in Table 4. The quantities of NMP and ACTY116 were kept constant while the PLGA quantity was varied to make the ratios of PLGA: ACTY116 range from 0:1 to 15:1. The different formulations were filled into 7 mL vials and sealed with rubber stoppers. The filled vials were autoclaved under 121 °C for 8 min and then tested for assay values.

Evaluation of the impact of vial diameter on the ACTY116 stability in pLAIs was performed using Formulation F11 (Table 4). The suspension was filled into glass vials with different inner diameters (4, 10, and 19 mm) and sealed with rubber stoppers. The filled vials were autoclaved under 121 °C for 8 min and then tested for assay value.

Evaluation of the impact of residual oxygen in the headspace on ACTY116 stability in pLAIs was performed using formulation F11, as shown in Table 4. The formulation was filled into 10 mm-diameter vials, and residual oxygen in the headspace was controlled between 0.1% and 5.0%. The filled vials were autoclaved under 121 °C for 8 min and then tested for assay values.

#### 2.3.4. Stability Study of ACTY116 pLAI In Situ Depot Gel

The stability evaluation of the selected ACTY116 pLAI in situ depot gel was performed using formulation F11, as shown in Table 4. The formulation suspension was filled into 4 mm-diameter vials, headspace oxygen was controlled to be not more than 0.1% and sealed with rubber stoppers. The samples for stability study were stored under the harsh condition (40 °C/75% RH) and the long-term storage condition (25 °C/65% RH), respectively. Critical quality attributes such as assay, headspace oxygen, viscosity, and polarizing microscope observation were tested at predetermined time intervals.

Viscosity of the ACTY116 in situ depot gel was determined with a digital rotary viscometer, 3.0 rpm for 2 min [21]. The ACTY116 in situ depot gel was dropped onto the glass slide for polarizing microscopic observation [22,23].

#### 2.3.5. Conformational Stability by Computer Molecular Dynamics (MDs) Simulations

The initial structure of ACTY116 with an alpha helix conformation was constructed using SYBYL-X 1.3 [24], and the copolymer PLGA was generated by AMBER 16 [25] employing GAFF2 force fields. The 12 units of ACTY116 were dissolved in water and set to periodic boundary within 10 Å, then heated to 373.15 K, and subjected to 100 ns production MDs simulations using the AMBER 16 package with ff14SB [26]. After density adjustment and equilibrium, the complex obtained a stable conformation while the root mean square deviation (RMSD) fluctuated steadily over 20 ns. Similarly, the ensemble of the complex with ACTY116 and PLGA (molar ratio of ACTY116:PLGA is about 1:2 in F11) was manually constructed, then dissolved in NMP within 10 Å to the edge of the periodic boundary; after density adjustment and equilibrium using AMBER 16, MDs simulations were performed under 373.15 K for 100 ns by AMBER 16. The average structure was extracted from the latest 20 ns for analysis. All visualisations were presented using Pymol 2.5 [27].

#### 2.3.6. Evaluation of ACTY116 Formulations for Pharmacological Activity in Mice with Cardiac Hypertrophy Induced by Norepinephrine

Peptide ACTY116 was designed to treat cardiac hypertrophy (CH). An animal model of cardiac hypertrophy in mice induced by norepinephrine (NE) has been reported and was used in this study to evaluate the pharmacological action of ACTY116 (F1) solution and pLAI in situ depot gel (F11) [28]. BALB/C mice were randomly divided into 5 groups (6 mice/group) as follows:

Group 1: Vehicle-control mice received subcutaneous injection of a solution containing 5% glucose and 0.1% vitamin C, 20 mL/kg/d, bid, for 14 successive days.

Group 2: NE-treated mice received subcutaneous injection of NE (3.0 mg/kg/d) bid, for 14 successive days to induce cardiac hypertrophy.

Group 3: NE-treated mice also received subcutaneous injection of ACTY116 solution at a dose of 1.0 mg/kg, bid, for 14 successive days.

Group 4: NE-treated mice also received subcutaneous injection of ACTY116 pLAI at a dose of 7 mg/kg, once a week on day 0 and day 7.

Group 5: NE-treated mice also received subcutaneous injection of ACTY116 pLAI at a single dose of 14 mg/kg on day 0.

**Figure 3:**
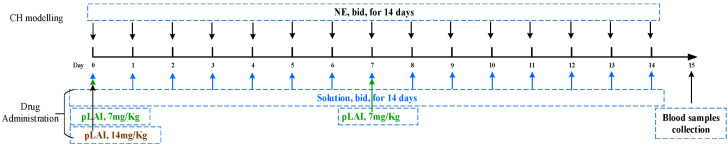
CH modelling and drug administration.

The dose regime is illustrated in Figure 3.

At the day 15, blood samples for all mice were collected from the retrobulbar venous plexus, and the plasma was separated by refrigerated centrifuge at 4 °C. The biomarker for cardiac hypertrophy, i.e., NT-Pro-brain natriuretide (NT-pro BNP) was analysed with an ELISA kit [29,30]. The heart tissues were harvested from mice, and cardiac morphology was then observed and recorded. Subsequently, the heart weight, body weight and tibial length were measured [31]. The heart weight to body weight ratio (HW/BW) and heart weight to tibial length (HW/TL) were then calculated and evaluated with statistical analysis. The heart tissues were dissected, and part of these tissues were fixed in 4% buffered formalin for 48 h, then dehydrated, embedded in paraffin, and sectioned. BNP and β-MHC immunohistochemical analysis were performed: diaminobenzidene chromogenic reaction was performed after BNP and β-MHC antibody incubation, respectively, and the positive expression of BNP and β-MHC was brownish yellow. H&E and WGA staining were performed, and the images were scanned and observed using a slide scanner [32,33,34]. Thereafter, the pathological changes and the cross-sectional areas of cardiomyocytes in heart tissues were measured by using ImageJ software (version 1.8.0.172, NIH, Bethesda, MD, USA).

#### 2.3.7. In Vitro Release Testing for ACTY116 PGLA In Situ Depot Gel Formulations

Impact of both quantity and type of PLGA in the in situ depot gels on in vitro ACTY116 release was performed using different formulations. Formulations with different quantities of PLGA (L:G = 75:25, MW: 10,000–20,000) are provided in Table 5 while formulations with different type of PLGA are listed in Table 6. An accelerated method was used to test in vitro release of ACTY116 PLGA in situ depot gel. An elevated temperature around the glass transition (Tg) of PLGA was adopted as a method to accelerate peptide release from the PLGA matrix. When the PLGA is close to/above Tg, the polymer is in the rubbery state, where mobility of the polymer results in significant acceleration of peptide release via diffusion, and hydration/degradation is also accelerated when compared to the glassy state [35,36]. An accelerated method by elevating the temperature was used to test in vitro release of ACTY116 pLAI in situ depot gel. Briefly, about 30–50 mg of ACTY116 pLAI in situ depot gel was pipetted into the 20 mL vial dissolution device containing 20 mL purified water as release medium at 50 ± 1 °C and under 100 rpm continuous shaking conditions. An aliquot of 200 µL of the release medium was sampled at 2, 5, 24, 48, 72, 96, 120, 144, and 168 h, respectively, and the released peptide ACTY116 was analysed by the bicinchoninic acid (BCA) method [37].

#### 2.3.8. Pharmacokinetics of ACTY116 Solution and pLAI In Situ Depot Gels in Rats

ACTY116 solution (F1) was injected subcutaneously in rats for pharmacokinetic (PK) behaviour as an immediate release formulation. Six (6) rats received ACTY116 solution at a dose of 1.0 mg/kg, bid, and blood samples were collected from the retrobulbar venous plexus at minutes 10, 20, 40, 60, 90, 120, and 180 after subcutaneous administration; then, plasma was separated by refrigerated centrifuge, and the level of ACTY116 was analysed with a LC-MS/MS method.

Formulations (F14, F15, and F16 in Table 5) were used to evaluate the impact of PLGA quantity on in vivo pharmacokinetics in rats. Eighteen rats were randomly divided into 3 groups (6 rats/group), and each group of rats received different ACTY116 pLAI formulations at a single SC dose of 14 mg/kg. The blood samples were collected from the retrobulbar venous plexus at hours 1, 2, 4 and on days 1, 3, 5, 7, 9, 11, 13, 15, 17, 19, 21, 23, 25, and 27 post subcutaneous injection; then, plasma was separated by refrigerated centrifuge, and ACTY116 was analysed using a LC-MS/MS method.

Formulations (F17, F18, F19, and F20 in Table 6) were used to evaluate the impact of different types of PLGA on in vivo pharmacokinetics in rats. Eighteen rats were randomly allocated to 3 groups (6 rats/group). Each group of rats received ACTY116 pLAI formulated with a different PLGA type at a single SC dose of 14 mg/kg. The blood samples were collected from the retrobulbar venous plexus at hours 1, 2, and 4 and on days 1, 3, 5, 7, 9, 11, 13, 15, 17, 19, 21, 23, 25, 27, 29, 31, 33, and 35 post subcutaneous injection; then, plasma was separated by refrigerated centrifuge, and ACTY116 was analysed with a LC-MS/MS method.

## 3. Results

### 3.1. Stability Evaluation of Different Dosage Forms

**Figure 4:**
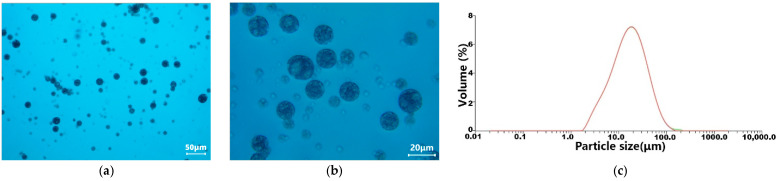
The polarizing microscope observation of ACTY116 microspheres: (**a**) 20×, (**b**) 80×; (**c**) particle size distribution.

The microscope observation of ACTY116 microspheres are displayed in Figure 4a,b. Particle size distribution (Figure 4c) of the microspheres was tested, and the results showed that D (10) was 5.12 µm, D (50) was 16.6 µm, D (90) was 38.3 µm, and D (4,3) was 21.9 µm. Figure 4c depicts the results of three measurements, represented by curves of three different colors. The repeatability of the three measurements is good, the curves almost completely overlap. Encapsulation efficiency was 29.8%.

#### 3.1.1. Chemical Stability Evaluation of Different Dosage Forms

Stability data in Table 7 showed that the assay of ACTY116 in microsphere (F2) decreased dramatically to 86.78% after preparation. However, the assay of ACTY116 in solution (F1) and pLAI (F3) were kept constant after preparation.

Short-term stability under 40 °C showed that the assay in solution (F1) decreased to 86.75% after 5 days and 67.39% after 10 days while the assay in microspheres (F2) decreased to 79.54% after 5 days and 58.46% after 10 days. Surprisingly, the assay was kept at 99.12% after 5 days and 98.23% after 10 days for ACTY116 pLAI in situ depot gel (F3). Data suggested that peptide ACTY116 appears to be much more stable in the pLAI in situ depot gel formulation than that in a solution or microspheres.

Stability comparison results of different dosage forms suggested that neither solution nor the microspheres were stable enough for further development, and non-aqueous in situ depot gel formulations have the potential to maintain the chemical stability of ACTY116; thus, formulation optimization of ACTY116 pLAI in situ depot gel was further conducted.

#### 3.1.2. Secondary Structure of ACTY116 Determined by Circular Dichroism

**Figure 5:**
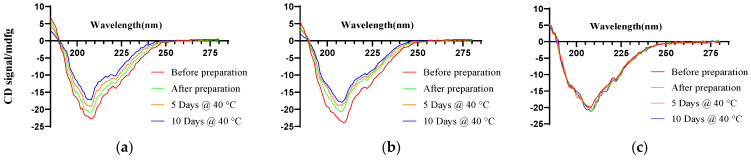
The CD spectra of different ACTY116 dosage forms: (**a**) ACTY116 solution (F1); (**b**) ACTY116 microspheres (F2); (**c**) ACTY116 pLAI in situ depot gel (F3).

A secondary structure of a peptide is commonly evaluated by circular dichroism (CD) [38,39]. The circular dichroism (CD) spectrum reveals a prominent negative absorption peak at 208 nm for ACTY116, indicative of its typical α-helical characteristics. Before preparation, the CD spectrum of ACTY116 in solution (Figure 5a) displayed a prominent negative band at 208 nm with a very strong intensity. However, following preparation, the characteristic band at 208 nm gradually diminished. Moreover, after storage at 40 °C for 5 and 10 days, the CD signals decreased further. The CD spectra of ACTY116 in microspheres (Figure 5b) showed a similar pattern after preparation and storage under 40 °C. Because organic solvent evaporation under 50 °C for 4 h was conducted, the process may cause the secondary structure change of peptide ACTY116 during preparation. When stored under 40 °C, the peptide could be aggregated, which may lead to the change of the peptide secondary structure. However, as can be seen (Figure 5c), the CD spectra of ACTY116 in pLAI in situ depot gel have no change in all samples. The CD spectra indicated that the secondary structure of ACTY116 in solution and microspheres had been altered after preparation and storage in high-temperature conditions for several days; however, the characteristic band at 208 nm remained unchanged for samples from different conditions in pLAI in situ gel. Thus, the secondary structure of ACTY116 can be well protected using the pLAI in situ gel formulation approach.

Stability comparison results of different dosage forms suggested that neither solution nor microspheres were stable enough for further development, whereas in situ depot gel formulations have the potential for a better stability; thus, formulation optimization of ACTY116 pLAI in situ depot gels were further conducted.

### 3.2. Formulation Optimization of ACTY116 pLAI In Situ Depot Gels

ACTY116 in situ depot gel formulation variables evaluated in this study include quantity of NMP and PLGA, vial diameter, and residual oxygen in headspace.

**Figure 6:**
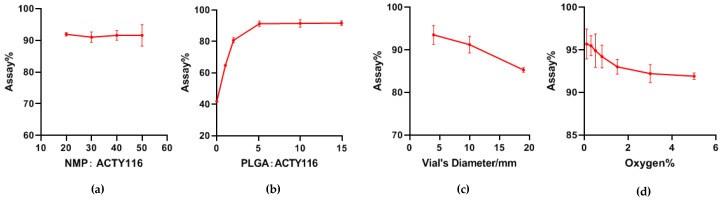
The results of variable evaluation: (**a**) evaluation of impact of NMP quantity on ACTY116 stability in pLAIs (F4–F7); (**b**) evaluation of impact of PLGA quantity on ACTY116 stability in pLAIs (F8–F13); (**c**) evaluation of impact of surface area of liquid–air interface (three sizes of vials with diameters of 4 mm, 10 mm, and 19 mm) on ACTY116 stability in pLAIs; (**d**) evaluation of impact of residual oxygen in the headspace on ACTY116 stability in pLAIs. Data are represented as mean ± SD (*n* = 4).

NMP was used as a non-aqueous solvent in the formulation, which is critical for dissolving PLGA. We varied the ratio of NMP to ACTY116 to explore the impact of NMP on peptide stability. As shown in Figure 6a, an increase in the ratio of NMP to ACTY116 from 20:1 to 50:1 (F4–F7) did not show a negative impact on the assay value of ACTY116, which means that NMP may not impact ACTY116 stability.

PLGA is a controlled-release polymer, which is critical for the long-acting effect of the formulation. The effects on its stability of formulations with different ratios of PLGA (F8–F13) to ACTY116 have been evaluated after terminal sterilization. As shown in Figure 6b, ACTY116 is degraded rapidly without PLGA in the formulation (F8), and only 42% of the assay remained after being treated at 121 °C for 8 min. When the ratio of PLGA to the peptide increased, the assay value of the peptide increased correspondingly after thermal treatment. When the ratio of PLGA to ACTY116 increased 5:1 (F11), the assay of ACTY116 remained above 90% after terminal sterilization. However, increases in the ratios greater than 5:1 did not show further protection of ACTY116 from thermal degradation. Formulation F13 had the highest ratio of PLGA to ACTY116, i.e., 15:1, and the assay of the peptide after terminal sterilization was 91.6%, which is similar to that of F11. The results suggested that PLGA should have a protective function for ACTY116 during terminal sterilization, and a minimal ratio of PLGA to ACTY116 (5:1) is required for maximal protection.

The presence of liquid–air interfaces in peptide/protein pharmaceuticals is known to negatively impact product stability. Vial diameter is a formulation variable because the size has an impact on the surface area of the liquid–air interface. Three sizes of vials with diameters of 4 mm, 10 mm, and 19 mm were filled with the formulation F11 and used in the surface area study. As seen in Figure 6c, the assay value of ACTY116 F11 decreased rapidly with an increase in the vial diameter. When the diameter of the vial was 4 mm, the assay value of ACTY116 was 93.5% after being treated at 121 °C for 8 min while the assay decreased to 85.3% with the vial diameter of 19 mm. Obviously, the vial diameter has a significant impact on degradation of ACTY116, and small diameters reduced the thermal degradation of the peptide, which may be related to the smaller area of liquid–air interface.

Impact of oxygen levels in the headspace of ACTY116 assay has been studied, and the results are provided in Figure 6d. As can be seen, the higher the oxygen level in the headspace is, the lower the assay value would be. When the headspace oxygen was at about 5%, the assay value of ACTY116 decreased to 91.9% after being treated under 121 °C for 8 min while if the oxygen level was reduced to 0.1%, the assay of ACTY116 was 95.7%, which is significantly higher than that of headspace oxygen at 5%. Thus, the oxygen level in the headspace should be controlled to be as low as possible to stabilise the ACTY116 in the pLAI formulation.

### 3.3. Stability Study of ACTY116 pLAI In Situ Depot Gel

The results demonstrated the excellent stability of ACTY116 pLAI following terminal sterilization at 121 °C for 8 min, with the assay value of ACTY116 remaining as high as 98.6%. Storage at 25 °C/60% RH for up to 6 months and at 40 °C/75% RH for 3 months did not lead to any noticeable changes in assay values. Moreover, no discernible alterations in appearance, headspace oxygen levels, or viscosity were observed (see Table 8).

**Figure 7:**
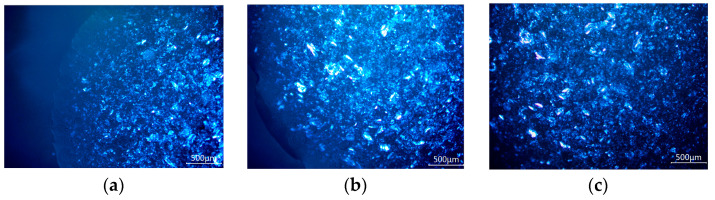
Polarised microscopic observation of ACTY116 in situ depot gel: (**a**) month 0; (**b**) long-term stability: month 6; 25 °C/60% RH; (**c**) accelerated stability: month 3; 40 °C/75% RH.

The polarised microscope observation (30×) of ACTY116 pLAI in situ depot gel is presented in Figure 7; shiny particles were observed in the microscopic image. Since ACTY116 was the sole component exhibiting doubly refracting (optically anisotropic) properties in the pLAI formulation, resulting in interference effects under the polarised light microscope, the shiny particles seen in Figure 7 were identified as ACTY116 peptide crystals. The stability study indicated that the ACTY116 crystals observed through the polarizing microscope in the pLAI formulation remained unchanged throughout the study period.

In summary, stability data for ACTY116 pLAI in situ depot gel formulation indicated that the formulation is stable and suitable for further in vivo pharmacodynamics and pharmacokinetics studies.

### 3.4. Conformational Stability Evaluation by Computer Molecular Dynamics (MDs) Simulations

**Figure 8:**
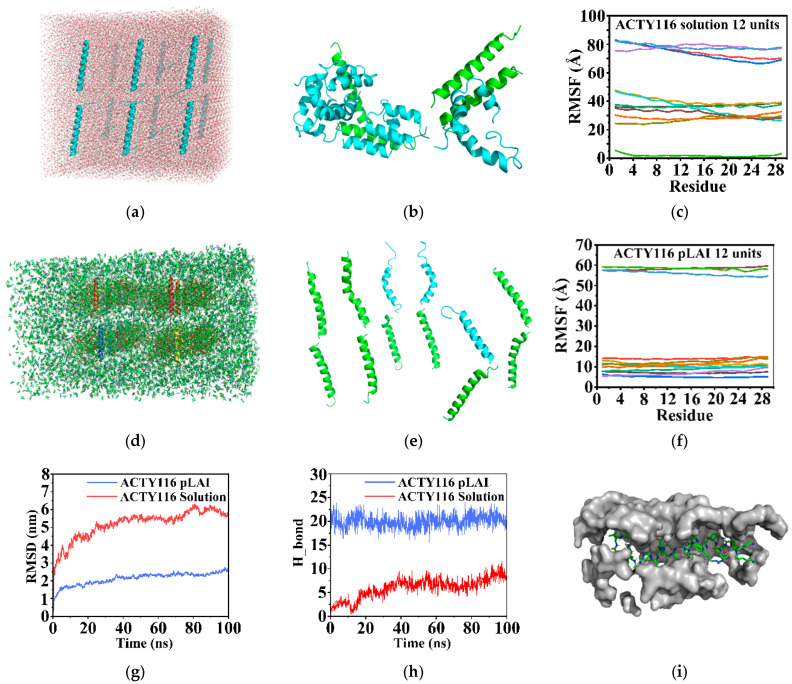
(**a**) Ensemble of ACTY116 initial state solvated in water; (**b**) MDs simulations under 373.15 K for 100 ns in water; (**c**) root mean square fluctuation (RMSF) value of ACTY116 solvated into water under 373.15 K; (**d**) ensemble of ACTY116 initial state complex of ACTY116 and PLGA solvated into NMP; (**e**) MDs simulations under 373.15 K for 100 ns in PLGA and NMP system; (**f**) root mean square fluctuation (RMSF) value of ACTY116 solvated into PLGA and NMP system under 373.15 K; (**g**) root mean square fluctuation (RMSF) value of ACTY116 solvated into water under 373.15 K for 100 ns; (**h**) RMSF value of ACTY116 solvated into PLGA and NMP system under 373.15 K for 100 ns; (**i**) visualisation of ACTY116 unit complexed with PLGA (gray part) around 10 Å distance.

The initial structure of ACTY116 with an alpha helix conformation was established by SYBYL-X 1.3. An ensemble with 12 ACTY116 units was constructed and solvated with water (Figure 8a). After conducting 100 ns of MD simulations, eight units of ACTY116 experienced a conformational change from the middle of the helix when the system was heated to 373.15 K (Figure 8b). To investigate the conformational dynamics of ACTY116, the root mean square fluctuation (RMSF) value during 100 ns of MD simulations were analysed (Figure 8c), in which eleven units of ACTY116 became looser, and the results showed that Lys1-Lys2 and Asn27-Val29 exhibited an obvious change in the displacement of amino acids at C- and N-terminals. The sequences Lys1-Asn6 and Lys24-Val29 were presented in a loop, and seven units of ACTY116 in the middle area (Phe11-Asp16) experienced a secondary structure change. Most fluctuations were observed in Lys1-Asn6, which may be due to the temperature increase that makes the thermodynamic trajectory of amino acids larger. Interestingly, when ACTY116 was complexed with PLGA and solvated into NMP (Figure 8d), only three units of ACTY116 had a slight change in their terminal while others still maintained the same secondary structure (Figure 8e) during 100 ns of MDs simulations. To investigate the conformational dynamics of ACTY116 in PLGA and the NMP system, RMSF values during 100 ns of MDs simulations were analysed (Figure 8f), three units of ACTY116 became looser, and the sequences Lys1-Thr4 and Leu23-Val29 were presented in a loop, while other ACTY116 units maintained a rigid structure during the 100 ns MDs simulation.

Figure 8g indicates that the ensemble with 12 units reached equilibrium from the RMSD value. The average structure was extracted from the latest 20 ns while the system reached equilibrium. ACTY116 complexed with PLGA became more rigid due to the steric hinderance, which has a much lower RMSD value compared to ACTY116 solution. There were some shifts in Lys1-Thr4 and Lys24-Val29 of the ACTY116 secondary structure from alpha-helix to random coil in the water system. Figure 8h indicates that the average hydrogen bonds formed between ACTY116 and different solvent systems during 100 ns MD simulations, and 20 hydrogen bonds were formed between ACTY116 and NMP or PLGA, which shows a higher hydrogen bond value compared to the water system when heated to 373.15 K. In the PLGA and NMP systems, amino acids (such as Lys2, Asn6, Lys15, Asn22, Tyr26, and Leu28) formed hydrogen bonds. That is the reason nine units of ACTY116 remain a rigid structure under 373.15 K for 100 ns MDs simulations, and the hydrogen bonds were kept steady during the whole 100 ns under 373.15 K. However, hydrogen bonds in the water system showed an increasing trend during 100 ns under 373.15 K, which means the secondary structure of ACTY116 changed under high temperature, more amino acids were exposed to water, and more hydrogen bonds were formed between ACTTY116 and water. Figure 8i shows the visualisation of peptide ACTY116 in which it was surrounded by PLGA during the 100 ns MDs simulations.

### 3.5. Pharmacological Effect of ACTY116 Solution and pLAI In Situ Depot Gels on Cardiac Hypertrophy Induced by NE in Mice

**Figure 9:**
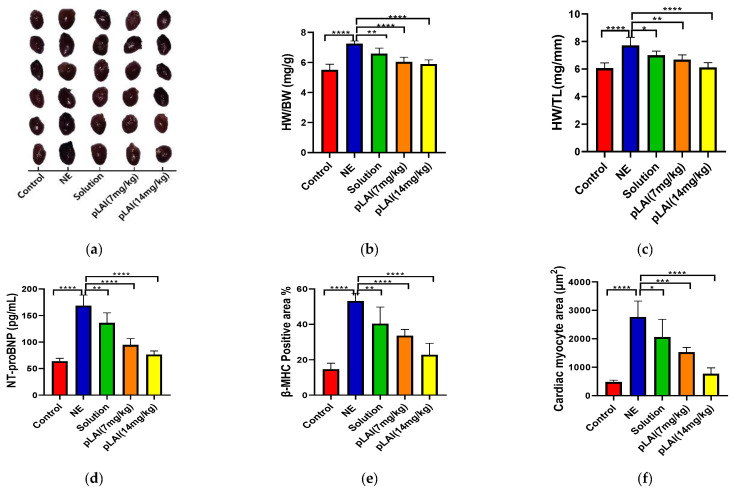

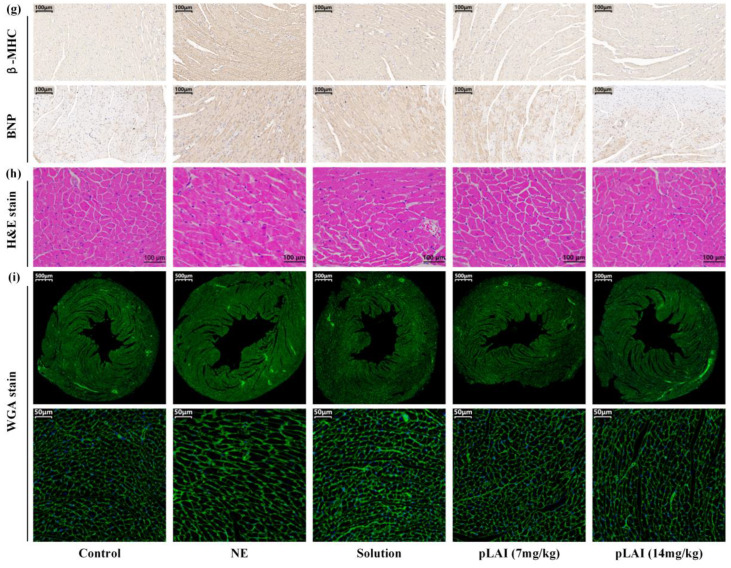
Evaluation of pharmacological activity on ACTY116 solution and pLAI formulations: (**a**) heart sizes; (**b**) heart weight to body weight (HW/BW) ratios; (**c**) heart weight to tibial length (HW/TL) ratios; (**d**) serum levels of NT-proBNP; (**e**) quantitative analysis of β-MHC expression; (**f**) quantitative analysis of myocyte cross-sectional areas in heart tissue sections with HE staining; (**g**) representative images of β-MHC (upper) and BNP (lower) expression (positive expression is brownish yellow); (**h**) representative images of H&E staining of heart tissues; (**i**) representative images of WGA staining of heart tissues. *n* = 6 in each group, * *p* < 0.05, ** *p* < 0.01, *** *p* < 0.001, **** *p* < 0.0001.

Cardiac hypertrophy in mice can be induced by subcutaneous injection of NE for 2 weeks. Pharmacological results showed that many symptoms related to cardiac hypertrophy can be observed in mice, including significant changes in anatomy of the heart and level of BNP, NT-proBNP, and β-MHC. As seen in Figure 9a, NE-treated mice had larger hearts visually than those of mice in the control group. Figure 9b shows that the heart sizes (heart weight (mg)/body weight (g) (HW/BW)) in the ACTY116-solution group appeared similar to those in NE-treated mice. When ACTY116 pLAI in situ depot gel was injected, both dose regimes (i.e., 7 mg/kg once a week for two weeks or 14 mg/kg once every two weeks) showed smaller heart sizes than for the NE-treated group and the ACTY116-solution group by visual observation. The heart weight in the control group was 5.5 mg/g mice while it was increased to 7.2 mg/g body weight after being treated with NE, bid, for 2 weeks, which is a significant increase compared to the control group (*p* < 0.0001). However, if NE-treated mice were given ACTY116 solution at the same time, bid, for 14 days, the heart weight tended to decrease compared with the NE-treated group. At the end of the 14-day treatment, the heart weight for the ACTY116-solution group was 6.6 mg/g body weight, which is significantly lighter than that of the NE-induced group (*p* < 0.01). The results suggested that the ACTY116 solution showed certain pharmacological effect on mice with cardiac hypertrophy. When NE-treated mice were injected with the ACTY116 pLAI formulation at 7 mg/kg, once a week for 2 weeks, the heart weight of mice at day 15 was 6.0 mg/g body weight, which is significantly different in comparison to NE-treated mice (*p* < 0.0001). Another group of NE-treated mice were injected with ACTY116 pLAI formulation at 14 mg/kg, single dose, and the heart weight of the mice at day 15 was 5.9 mg/g body weight. The change in heart weight of mice at the 14 mg/kg dose level was significantly different from that of the NE-treated group. Similar results were obtained for the heart weight (mg)/tibial length (mm) (HW/TL) (Figure 9c) from the above studies. The results indicated that the ACTY116 pLAI formulation has a long-acting effect, and the pharmacological action is superior than that of solution.

A biomarker, i.e., NT-proBNP, in serum is another indicator for cardiac hypertrophy. Two weeks after treatment, the level of NT-proBNT very significantly increased in the NE-treated group (168.43 pg/mL) compared to the control (63.96 pg/mL) group (*p* < 0.0001). Three ACTY116-treated groups showed a significant decrease in serum NT-proBNP (Figure 9d). The serum level of NT-proBNP after the 2-week treatment was 136.12 pg/mL for ACTY116 solution (*p* < 0.01), 94.61 pg/mL for 7 mg/kg once a week for 2 weeks (*p* < 0.0001), and 76.39 pg/mL for 14 mg/kg, single dose (*p* < 0.0001). Similarly, treatment with ACTY116 pLAI obviously decreased β-MHC and BNP expression. The darker the brownish yellow colour, the higher the β-MHC and BNP expression. Results showed that the level of β-MHC expression (Figure 9e) significantly increased in the NE-treated group compared with the control group; the average β-MHC-area of NE-treated group was 53.2%, and 14.6% for the control group (*p* < 0.0001). Three ACTY116-treated groups showed a significant decrease in β-MHC expression. The average β-MHC-positive area was 40.3% for ACTY116 solution (*p* < 0.01) after 2-week treatment, 33.6% for 7 mg/kg once a week for 2 weeks (*p* < 0.0001), and 22.8% for 14 mg/kg, single dose (*p* < 0.0001). Results of BNP expression showed a similar trend, which further proves the pharmacological activity of ACTY116; ACTY116 pLAI in situ depot gel has long-acting effect for cardiac hypertrophy. Representative images of β-MHC (upper) and BNP (lower) expression are shown in Figure 9g.

To investigate the effects of ACTY116 on pathological changes of the heart tissue, H&E (Figure 9h) staining and WGA (Figure 9i) staining were performed, and the quantitative analysis of myocyte cross-sectional areas in heart tissue sections were calculated by software ImageJ (Figure 9f). Compared to the control group, NE markedly increased the cross-sectional area of cardiomyocytes; the mean area increased from 482.7 µm^2^ to 2768.2 µm^2^. However, treatment with ACTY116 pLAI could significantly decrease the cardiomyocyte cross-sectional area, compared to the NE group: 1530.6 µm^2^ for 7 mg/kg once a week for 2 weeks (*p* < 0.001) and 773.1 µm^2^ for 14 mg/kg, single dose (*p* < 0.0001). The pharmacological activity is dose-dependent, in which a higher dose (14 mg/kg) showed stronger pharmacological activity than a lower dose (7 mg/kg).

The pharmacodynamics study confirmed the successful establishment of a cardiac hypertrophy mouse model induced by NE. Moreover, the pLAI formulation exhibited superior pharmacological efficacy compared to the ACTY116 solution, allowing for a reduction in injection frequency from twice daily to once weekly or even biweekly.

### 3.6. Effect of PLGA Copolymer on In Vitro Drug Release from pLAI In Situ Depot Gels

**Figure 10:**
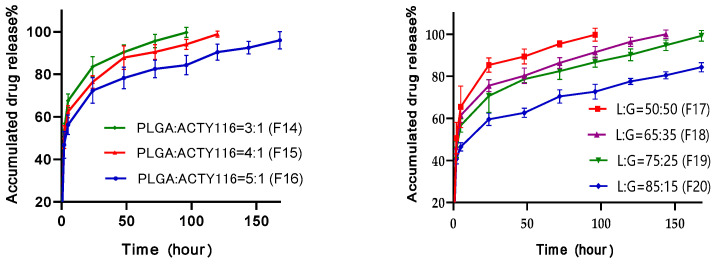
Effect of PLGA copolymer on in vitro drug release from pLAI in situ depot gels: (**a**) impact of PLGA quantity on ACTY116 in vitro release; (**b**) impact of type of PLGA on ACTY116 in vitro release.

Impact of quantity of PLGA on in vitro drug release was studied on formulations with PLGA:ACTY116 ratios of 3:1 (F14), 4:1 (F15), and 5:1 (F16), respectively. When PLGA:ACTY116 ratio was 3:1 (F14), the accumulated drug release was 83.6% at 24 h, and complete release was found at 96 h (Figure 10a). When the ratio of PLGA:ACTY116 increased to 4:1 (F15) and 5:1 (F16), the drug release decreased accordingly, and complete release was 120 h for F15 and 168 h for F16, respectively. Hence, increase in PLGA quantity may significantly decrease the in vitro release rate for ACTY116.

Three types of PLGAs have been formulated with ACTY116 and NMP, including a lactide:glycolide (L:G) ratios of 50:50, 65:35, 75:25, and 85:15, respectively. As shown in Figure 10b, when the L:G ratio was 50:50, the accumulated drug release from F16 was 85.4% at 24 h and 95.5% at 72 h. When the L:G ratio was increased to 65:35 (F18), the drug release rate was slowed down. The accumulated drug release was 75.5% at 24 h and 86.4% at 72 h, respectively. By increasing the L:G ratio to 75:25 (F19), the accumulated drug release was only 70.6% at 24 h and 82.4% at 72 h, respectively. Further increasing the L:G ratio to 85:15 (F20) led to the slowest drug release rate in vitro. The results indicated that PLGA should be effective to control the drug release rate, and changes in L:G ratios can be used to formulate the ACTY116 pLAI in situ depot gel with different drug release rates.

Therefore, both quantities of PLGA and different types of PLGAs in formulation can change the release rate of ACTY116 from an in situ depot gel, so a pLAI formulation with an ideal release rate can be designed with varying quantities and types of PLGAs in the formulation.

### 3.7. Pharmacokinetics of ACTY116 Solution and pLAI Formulations in Rats

The ACTY116 solution (F1) and pLAI formulations with different quantities and types of PLGA were subcutaneously administrated in rats to study pharmacokinetic behaviour. The pharmacokinetic parameters in Table 9 show that ACTY116 pLAI formulations could significantly decrease drug clearance (Cl) from 2167.2 to less than 559.1 mL/h/kg compared to the solution, the half-life and the mean residence time (MRT) was extended from less than 1 h up to hundreds of hours, and the area under curve (AUC) was increased about 100 times, which means the pLAI formulations could reach the long-acting goal.

**Figure 11:**
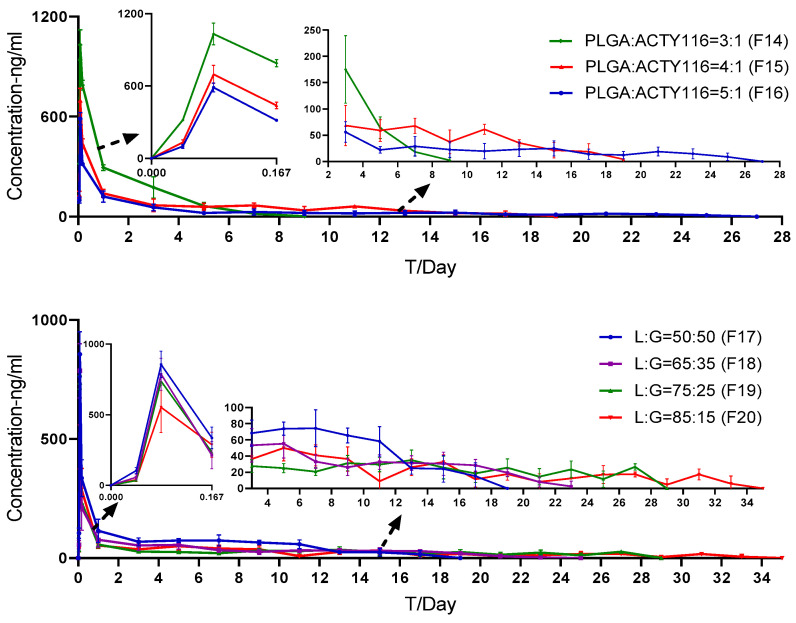
Evaluation of different doses on ACTY116 the in vivo PK profile.

The effect of PLGA quantity (formulations are listed in Table 5) on pharmacokinetics was studied. As displayed in Table 9, an increase in the PLGA:ACTY116 ratio resulted in a decrease in Cmax from 1014.3 ng/mL (F14) to 587.8 ng/mL (F16), while extending the half-life from 34.1 h (F14) to 224.4 h (F16) and the MRT from 47.1 h (F14) to 161.7 h (F16). The pharmacokinetic parameters indicated that different PLGA:ACTY116 ratios have an impact on in vivo release of ACTY116 and thus pharmacokinetic profiles in rats. Longer MRTs and half-lives were obtained with a higher PLGA:ACTY116 ratio (Figure 11).

The effect of different types of PLGA on pharmacokinetics was also studied. As displayed in Table 10, an increase in the L:G ratio resulted in a decrease in C_max_ from 806.1 ng/mL (F17) to 552.8 ng/mL (F20), the half-life was extended from 111.4 h (F17) to more than 340 h (F19 and F20), and MRT was extended from 132.1 h (F17) to around 230 h (F19 and F20). The results indicated that different L:G ratios have an impact on in vivo release of ACTY116 and thus the pharmacokinetic profiles in rats. As the ratio of lactide increases (i.e., higher L:G ratio), the polymer degradation slows down due to the presence of a hydrophobic methyl group, and with the increase in lactide portion, the drug release was slower and the half-life and residence time were longer (Figure 11).

## 4. Discussion

The U.S. FDA (United States Food and Drug Administration) has approved around 20 long-acting injectables based on PLA/PLGA, primarily in microsphere form. While these products offer improved patient compliance and therapeutic benefits, their complex formulations and intricate processing contribute to high costs; moreover, the high drug load in microspheres makes any unforeseen changes in drug release characteristics risky, potentially leading to adverse effects. Microsphere products are sensitive to manufacturing changes, which may affect their physicochemical attributes, during in vitro and in vivo performance. Manufacturing challenges arise due to the proprietary process, resulting in difficulties achieving consistent high-quality products [39,40,41,42]. Peptide molecules often exhibit poor chemical stability, and the production process for microspheres complicates matters further: the peptide is dissolved in water and subjected to vigorous emulsification through high-shear mixing, and following the formation of a double emulsion, the organic solvent necessitates removal at elevated temperatures. Unfortunately, these harsh manufacturing processes serve to accelerate both peptide hydrolysis and oxidation [43,44]. In the early stages of formulation development, we compared two pLAI formulations: microspheres and in situ depot gel. Due to issues such as the complicated preparation process, low encapsulation efficiency, and the fact that ACTY116 showed poor stability in microspheres, we excluded microspheres as a pLAI formulation for ACTY116, and we concentrated on proceeding with the in situ depot gel formulation for further process optimization, molecular dynamics simulation, and pharmacological and pharmacokinetic studies in animals.

In pursuit of achieving extended drug release, this study has developed a long-acting injectable formulation utilising biodegradable polymers and an in situ depot gel, and the preparation process is straightforward, avoiding the use of harsh conditions such as high temperatures, high-shear mixing, or homogenization; furthermore, the maintenance of a water-free and oxygen-free environment ensures the chemical stability of peptide ACTY116. The results revealed superior stability of ACTY116 in the PLGA-based in situ depot gel compared to the solution or microspheres. Molecular dynamics (MDs) simulations highlighted increased rigidity of ACTY116 within the pLAI in situ depot gel, providing stability even under elevated temperatures due to enhanced hydrogen bonding, steric hindrance, and shielding effects. The long-acting release behaviour of in situ depot gel formulations was assessed both in vitro and in vivo. Water absorption starts immediately upon exposure of a PLGA matrix to water, or during in vivo administration. The release rate is initially diffusion-controlled, giving way to degradation/erosion at the end of release period. Diffusion occurs through water-filled pores within the PLGA matrix, which commonly exhibits classic tri-phasic release patterns including an initial burst (phase I) and a slow-release phase (lag time) associated with a diffusion-driven release (phase II), followed by a faster release (phase III) attributed to erosion [45]. Increase in PLGA quantity may significantly decrease the diffusion and erosion of the PLGA matrix, and it may lead to the decreased release rate. PLGA polymer composition (type) is another important factor contributing to degradation rate; it impacts the hydrophilicity, glass transition temperature (Tg), and hydration rate of the PLGA polymer. L:G ratio significantly influences solubility and degradation rate, impacting drug release. The glycolide is more hydrophilic due to the absence of the methyl side group rather than lactide, which means that a higher glycolide percentage causes more water uptake, and consequently an overall weight loss of the PLGA matrix occurs, and a faster degradation rate happens [46]. A pLAI formulation with an ideal release rate can be designed with varying the quantity and type of PLGA in the formulation. Pharmacological effects of different dosage forms also indicate that ACTY116 pLAI in situ depot gel could prolong in vivo release and reduce the dosing interval because of the long residence time.

However, there are still unresolved issues in our study. Similar to FDA-approved microsphere LAIs, our pharmacokinetic findings of ACTY116 pLAI in rats indicated a burst release, primarily due to the rapid drug diffusion from the surface of the in situ depot gel following subcutaneous injection [47,48]. Based on our current pharmacological and pharmacokinetic investigations, the burst release has not resulted in noticeable adverse effects. This might be attributed to the wider safety margin of ACTY116 or the limited duration of our observations. Prior to progressing to clinical trials, further assessments such as long-term toxicity studies are essential to evaluate whether the burst release could potentially lead to severe adverse effects. Currently, it appears that in situ depot gel, as a long-acting injectable formulation, is better suited for drugs with a broader safety margin, whereas it may not be suitable for drugs with a narrow therapeutic index.

## 5. Conclusions

The purpose of this article was to develop a polymeric long-acting injectable formulation of ACTY116 with high chemical and conformational stability to ensure a sustained and effective blood concentration of ACTY116 in vivo. We demonstrated that the ACTY116 pLAI in situ depot gel significantly reduces the frequency of ACTY116 administration, displaying a dose-dependent release duration. This formulation can achieve the desired long-acting effect as required, and it also exhibits superior in vivo therapeutic efficacy against cardiac hypertrophy in mice compared to ACTY116 solution.

## Figures and Tables

**Table 1:** Lyophilization process for ACTY116 PLGA microspheres.

Temperature (°C)	Duration (min)	Pressure (Pa)
−40	30	—
−40	180	—
−20	120	10–16
−20	270	10–16
0	180	10–16
25	120	10–16
25	600	10–16

**Table 2:** The gradient elution process.

Time/min	A (%)	B (%)
0	70	30
7	40	60
8	70	30
13	70	30

**Table 3:** Formulation compositions with different NMP quantities.

Formulation	F4	F5	F6	F7	Ingredient Function
ACTY116 (mg)	10	10	10	10	Active
PLGA (mg)	30	30	30	30	Control release polymer
NMP (mg)	200	300	400	500	solvent
NMP:ACTY116	20:1	30:1	40:1	50:1	——

**Table 4:** Formulation compositions with different PLGA quantities.

Formulation	F8	F9	F10	F11	F12	F13
ACTY116 (mg)	10	10	10	10	10	10
PLGA (mg)	0	10	20	50	100	150
NMP (mg)	200	200	200	200	200	200
PLGA:ACTY116	0:1	1:1	2:1	5:1	10:1	15:1

**Table 5:** Formulations with different PLGA:ACTY116 ratios.

	F14	F15	F16
ACTY116 (mg)	10	10	10
PLGA (L:G = 75:25) (mg)	30	40	50
NMP (mg)	200	200	200
PLGA:ACTY116	3:1	4:1	5:1

**Table 6:** Formulations with different PLGA types.

	F17	F18	F19	F20
ACTY116 (mg)	10	10	10	10
PLGA (mg)	50	50	50	50
NMP (mg)	200	200	200	200
PLGA type (L:G)	50:50	65:35	75:25	85:15
Molecular weight	7000–17,000	24,000–38,000	10,000–20,000	190,000–240,000

**Table 7:** ACTY116 assay change during preparation and short-term stability under 40 °C (*n* = 4).

	Before Preparation	After Preparation	5 Days @ 40 °C	10 Days @ 40 °C
Solution (F1)	100%	99.43 ± 1.6%	86.75 ± 2.3%	67.39 ± 2.1%
Microsphere (F2)	100%	86.78 ± 2.4%	79.54 ± 3.1%	58.46 ± 2.8%
pLAI in situ depot gel (F3)	100%	99.56 ± 0.8%	99.12 ± 1.7%	98.23 ± 1.3%

**Table 8:** Stability study results for ACTY116 in situ depot gel formulation F11.

Attribute	Initial	6 Months Under 25 °C/60% RH	3 Months Under 40 °C/75% RH
Assay%	98.6 ± 0.85	98.8 ± 1.32	98.3 ± 1.01
Headspace oxygen%	0.07 ± 0.02	0.05 ± 0.03	0.04 ± 0.01
Viscosity (cP)	115 ± 1.50	116 ± 1.83	113 ± 1.83
Appearance observed by polarizing microscope	Uniform shiny ACTY116 crystals in the suspension	Uniform shiny ACTY116 crystals in the suspension	Uniform shiny ACTY116 crystals in the suspension

**Table 9:** PK parameters of ACTY116 solution and ACTY116 pLAI formulations with different PLGA quantities.

PK Parameters	Solution (F1)	F14	F15	F16
C_max_ (ng/mL)	377.4 ± 20.3	1014.3 ± 388.3	709.5 ± 305.9	587.8 ± 289.9
AUC_last_ (h∙ng/mL)	226.9 ± 29.6	29,749.6 ± 12,223.4	28,072.2 ± 3732.3	21,015.0 ± 6374.0
AUC_INF_obs_ (h∙ng/mL)	234.1 ± 31.7	31,191.6 ± 116,423.0	31,790.4 ± 2771.5	26,369.1 ± 6491.9
HL_Lambda_z (h)	0.351 ± 0.021	34.1 ± 11.9	103.6 ± 27.1	224.4 ± 114.9
MRT_last_ (h)	0.796 ± 0.024	47.1 ± 10.8	124.6 ± 15.5	161.7 ± 23.0
C_last_ (ng/mL)	14.1 ± 4.0	25.2 ± 15.0	23.3 ± 8.7	16.4 ± 6.4
T_last_ (h)	2 ± 0	168.0 ± 30.4	416.0 ± 36.1	584.0 ± 24.8
Cl_F_obs (mL/h/kg)	2167.2 ± 276.2	507.5 ± 203.8	443.1 ± 37.9	559.1 ± 138.6

**Table 10:** Pharmacokinetic parameters of ACTY116 pLAI formulations with different PLGA types.

PK Parameters	F17	F18	F19	F20
C_max_ (ng/mL)	806.1 ± 295.0	736.6 ± 289.6	687.4 ± 246.2	552.8 ± 32.3
AUC_last_ (h·ng/mL)	27,601.0 ± 3464.0	21,177.7 ± 3186.0	20,035.8 ± 3527.0	23,157.4 ± 5337.8
AUC_INF_obs_ (h·ng/mL)	32,014.5 ± 5181.5	25,304.8 ± 3693.4	35,578.9 ± 11,920.6	31,563.1 ± 6039.8
HL_Lambda_z (h)	111.4 ± 42.4	156.7 ± 91.5	348.7 ± 121.6	340.9 ± 149.6
MRT_last_ (h)	132.1 ± 15.4	170.3 ± 12.4	232.8 ± 34.9	229.5 ± 34.0
C_last_ (ng/mL)	24.0 ± 16.2	16.0 ± 5.0	28.4 ± 12.8	17.1 ± 7.3
T_last_ (h)	392.0 ± 24.8	496.0 ± 36.1	632.0 ± 39.2	760.0 ± 24.8
Cl_F_obs (mL/h/kg)	445.4 ± 60.1	562.3 ± 74.9	445.9 ± 202.1	456.1 ± 80.1

## Data Availability

The datasets supporting the results reported in this article may be provided to researchers upon reasonable request. Such requests can be made by contacting the corresponding author (Xiaohui Li, lpsh008@aliyun.com).
